# Bismuth-Based Free-Standing Electrodes for Ambient-Condition Ammonia Production in Neutral Media

**DOI:** 10.1007/s40820-020-00444-y

**Published:** 2020-06-19

**Authors:** Ying Sun, Zizhao Deng, Xi-Ming Song, Hui Li, Zihang Huang, Qin Zhao, Daming Feng, Wei Zhang, Zhaoqing Liu, Tianyi Ma

**Affiliations:** 1 Institute of Clean Energy Chemistry, Key Laboratory for Green Synthesis and Preparative Chemistry of Advanced Materials, College of Chemistry, Liaoning University, Shenyang, 110036 People’s Republic of China; 2 Discipline of Chemistry, University of Newcastle, Callaghan, NSW 2308 Australia; 3 Key Laboratory for Green Synthesis and Preparative Chemistry of Advanced Materials, College of Chemistry, Liaoning University, Shenyang, 110036 People’s Republic of China; 4 School of Chemistry and Chemical Engineering, Guangzhou Key Laboratory for Environmentally Functional Materials and Technology, Guangzhou University, Guangzhou, 510006 People’s Republic of China

**Keywords:** N_2_ reduction, Bi_2_O_3_ nanoplate, Electrocatalysis, Free-standing

## Abstract

Electrocatalytic nitrogen reduction reaction is a carbon-free and energy-saving strategy for efficient synthesis of ammonia under ambient conditions. Here, we report the synthesis of nanosized Bi_2_O_3_ particles grown on functionalized exfoliated graphene (Bi_2_O_3_/FEG) via a facile electrochemical deposition method. The obtained free-standing Bi_2_O_3_/FEG achieves a high Faradaic efficiency of 11.2% and a large NH_3_ yield of 4.21 ± 0.14 $\upmu{\text{g}}_{{{\text{NH}}_{3} }}$ h^−1^ cm^−2^ at − 0.5 V versus reversible hydrogen electrode in 0.1 M Na_2_SO_4_, better than that in the strong acidic and basic media. Benefiting from its strong interaction of Bi 6*p* band with the N 2*p* orbitals, binder-free characteristic, and facile electron transfer, Bi_2_O_3_/FEG achieves superior catalytic performance and excellent long-term stability as compared with most of the previous reported catalysts. This study is significant to design low-cost, high-efficient Bi-based electrocatalysts for electrochemical ammonia synthesis.

**Electronic supplementary material:**

The online version of this article (10.1007/s40820-020-00444-y) contains supplementary material, which is available to authorized users.

## Highlights

- The Bi_2_O_3_ nanoplates homogeneously decorated free-standing exfoliated graphene (Bi_2_O_3_/FEG) was prepared by a facile electrochemical deposition method.
- The Bi_2_O_3_/FEG first used as nitrogen reduction electrocatalyst exhibits excellent electrocatalysis performance and stability for nitrogen reduction reaction in neutral media.
- The superior electrocatalytic nitrogen reduction activity is attributed to the strong interaction of the Bi 6*p* band with the N 2*p* orbitals, binder-free nature of the electrodes, and facile electron transfer through the graphene nanosheets.

**Electronic supplementary material:**

The online version of this article (10.1007/s40820-020-00444-y) contains supplementary material, which is available to authorized users.

## Graphical abstract

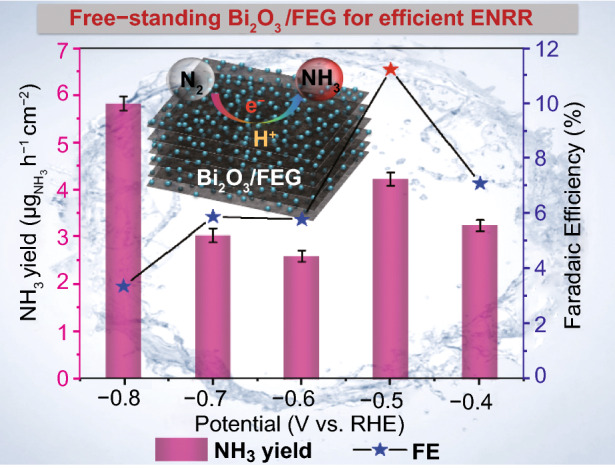

## Introduction

Ammonia (NH_3_) is considered to be a promising hydrogen energy carrier to alleviate the fossil fuel shortage and global climate change, for its high energy density, high hydrogen content, no carbon footprint and being easy to liquefy [1–3]. To date, the dominant method for industrial-scale NH_3_ synthesis still depends on the traditional Haber–Bosch process [4–6]. However, this energy-intensive and environmentally hazardous process consumes 2% of the global annual energy and leads to 1% of the world’s CO_2_ emission [7]. It is thus imperative to develop sustainable and economical techniques for NH_3_ synthesis at mild conditions.

The electrochemical nitrogen reduction reaction (ENRR) is regarded as an energy-saving and carbon-free process, which can synthesize NH_3_ at ambient conditions by utilizing renewable energy such as those from wind and solar [8–12]. Unfortunately, the scaled-up application of the ENRR process is seriously hampered by the low ammonia yields and Faraday efficiency due to the extremely weak N_2_ adsorption, the low solubility of N_2_ in aqueous electrolytes, and the sluggish cleavage of the N≡N bond [13]. Over the past 5 years, numerous metal-based materials, including metal [7, 14–18], metal oxides [19–21], metal sulfides [22, 23], metal nitrides [24], etc., have been developed as electrocatalysts for ENRR. Most of the above metal-based catalysts involving the metallic centers of Au [7], Ru [17], Ni [20], Mo [18, 22], etc., exhibited favorable activity for ENRR by elaborately designing the particle size, crystallinity, heteroatom doping, and/or vacancies. Despite the substantial progress, the ENRR still suffers from the major competitive hydrogen evolution reaction (HER). Therefore, the rational design of the active catalytic centers of efficient ENRR electrocatalysts that can efficiently reduce the large activation barrier of N≡N, accelerate its dissociation and most importantly suppress HER, is still a highly challenging but vitally important issue [25].

Bismuth-based catalysts are considered to be promising candidates for efficient ENRR in recent years [26–30]. Bi as a main group element with tunable *p*-electron density and intrinsic less reactive nature can selectively promote the reductive adsorption of N_2_ to form N_2_H^*^ without influencing the binding energy of the later intermediates, and also restrict the surface electron accessibility for effectively suppressing the competitive HER process [26–28]. Yan et al. demonstrated that overlaping of the Bi 6*p* bands and the 2*p* orbitals of the N atom both below and above the Fermi level can offer a lower free-energy change for the potential-determining step and better ENRR activity than typical transition metal catalysts [29]. Most of the Bi-based electrocatalysts for ENRR reported previously are mainly focused on the metallic bismuth (Bi^0^) [27–29]. It was reported that both the defect-rich Bi and 2D mosaic Bi nanosheets have been used for ENRR, and offered excellent selectivity in 0.2 M Na_2_SO_4_ (Faradaic efficiency (FE): 11.68% at − 0.6 V vs. RHE) [28] and 0.1 M in Na_2_SO_4_ (FE: 10.46 ± 1.45% at − 0.8 V vs. RHE) [31], respectively. As an abundant Bi-based material, Bi_2_O_3_ has received much attention in photocatalysis due to its unique electrical and optical properties [32–34]. It was also used as a precursor for the synthesis of other Bi-based materials [28]. However, bismuth oxide has hardly been reported for ENRR, with part of reason being that the semiconducting Bi_2_O_3_ shows poor charge transport, while the easy aggregation of Bi_2_O_3_ instead of nanosized status largely impairs the adsorption and reactivity to N_2_. Therefore, a robust support is necessary to enhance the conductivity, at the same time offer well dispersion of the Bi_2_O_3_ nanoparticles.

Functionalized exfoliated graphene (FEG) acts as an ideal 3D-conductive scaffold for supporting catalysts, which is constructed by ultrathin partially oxidized graphene nanosheets anchoring on the graphite substrate [35–37]. The FEG modified by oxygen-containing groups (–OH, –COOH) that provide active defect sites, can act as an excellent substrate for metal oxides deposition when using the metal ions as the precursor [38]. It is the high surface area and excellent electron conductivity of graphene nanosheets that can accelerate the charge transport and keep credible electric contact, resulting in the enormously improved catalytic efficiency [39–42]. In addition, polymer binder (e.g., Nafion) is widely used for attaching the electrocatalysts to the collectors, which seriously hampers the mass transport and obstructs the charge transmission, and therefore reduces the overall catalytic performance of the electrocatalysts [43, 44]. Therefore, the in situ growth of metal oxides on FEG with highly exposed active sites, superior electron conductivity, and large surface area is vitally important to improve the electrocatalytic performance. Based on the above analysis results, it is reasonable to assume that the composite electrocatalysts composed of well-coupled Bi_2_O_3_ and FEG can act as a promising candidate to promote the electrochemical reduction of N_2_ to NH_3_.

Herein, we developed a new type of ENRR catalyst by immobilizing Bi_2_O_3_ nanoplates onto the surface of FEG, to form the free-standing hybrid material (Bi_2_O_3_/FEG), which was then directly used as an electrode for ENRR in different electrolytes under ambient conditions. In this ENRR system, the competitive hydrogen evolution reaction (HER) is expected to be suppressed by the better intrinsic ENRR activity of Bi. Moreover, the highly conductive FEG was utilized to compensate for the semiconducting property of Bi_2_O_3_ and eliminate its aggregation. The as-prepared Bi_2_O_3_/FEG catalyst exhibits high ENRR activity with a high NH_3_ yield rate of 4.21 ± 0.14 $\upmu{\text{g}}_{{{\text{NH}}_{3} }}$ h^−1^ cm^−2^, and a favorable FE as high as 11.2% at − 0.5 V versus RHE in 0.1 M Na_2_SO_4_ electrolyte, out-performing most reported ENRR catalysts.

## Experimental

### Synthesis of the Functionalized Exfoliated Graphene (FEG)

The FEG substrate was prepared through an electrochemical exfoliation method according to our previous work with slight modification [45]. The exfoliation process was performed in a typical three-electrode cell using the graphite foil [GF, the exposed surface area is 1 × 1 cm^2^ (length × width)] as the working electrode, a platinum plate and a saturated calomel electrode (SCE) as the counter and reference electrodes, respectively. First, the GF was scanned between 0.5 and 1.7 V (vs. SCE) using cyclic voltammetry at 15 mV s^−1^ in 0.5 M K_2_CO_3_ electrolyte for ten cycles. Subsequently, the electrode was further treated at a constant potential of 1.8 V (vs. SCE) for 2 h in 0.5 M KNO_3_ aqueous solution to generate functional groups. Then, the electrode was potential dynamically scanned between − 1.0 and 0.9 V (vs. SCE) at 50 mV s^−1^ for 50 cycles in 3 M KCl electrolyte to recover the electrical conductivity. Finally, the FEG substrate was rinsed with deionized water and ethanol to remove the residuals.

### Synthesis of the nanosized Bi_2_O_3_ Modified FEG (Bi_2_O_3_/FEG)

Bi_2_O_3_/FEG was synthesized through an electrochemical deposition process. 0.3 g of bismuth chloride (BiCl_3_) was dissolved into 25 mL ethylene glycol (EG) and stirred at 25 °C for 1 h forming the electrolyte. The electrodeposition process was conducted in a two-electrode cell with a platinum plate as the counter electrode and a piece of FEG (1 × 1 cm^2^) as the working electrode. Bi_2_O_3_ nanoparticles were electrodeposited on FEG at a constant current density of 1 mA cm^−2^ at ambient temperature for 5 min. The as-synthesized product was washed with tetrahydrofuran and ethanol and subsequently dried in a vacuum oven at 70 °C for 12 h, finally obtaining free-standing Bi_2_O_3_/FEG.

### Electrocatalytic ENRR Measurements

Before ENRR tests, the Nafion 117 membrane was cut into small pieces and then treated with 3 wt% H_2_O_2_ water solution, deionized water, 1 mol L^−1^ H_2_SO_4_ and deionized water for 1 h at 80 °C, respectively. Finally, the obtained membrane was repeatedly rinsed until neutral pH was obtained and then was preserved in deionized water. Electrochemical measurements were performed in an airtight two-compartment cell at ambient conditions using an electrochemical workstation (CHI 760E), with Bi_2_O_3_/FEG or FEG as the working electrode, Pt foil as the counter electrode, and Ag/AgCl (filled with 3.5 M KCl solution) as the reference electrode. The potentials were converted to reversible hydrogen electrode (RHE) scale via calibration with the Nernst equation (*E*_RHE_ = *E*_Ag/AgCl_ + 0.059 pH + 0.205 V). The linear sweep voltammetry (LSV) curves were collected in 0.1 M Na_2_SO_4_ electrolyte saturated with ultrahigh Ar or N_2_ for 30 min at a sweep rate of 5 mV s^−1^, respectively.

### Determination of Ammonia

The concentrations of the synthesized NH_3_ in the electrolyte were measured by a colorimetric method using Nessler’s reagent as the color reagent. For this method, 10 mL electrolyte was mixed with 0.2 mL 50% seignette salt solution. Then 0.2 mL Nessler’s reagent was added and the mixture was allowed still for 10 min. Then the mixture was detected as the absorbance at 420 nm by a UV–Vis spectrometer (Shimadzu UV-2600). A standard curve of the Nessler’s reagent-based colorimetric method is constructed by measuring a series of absorbances for the reference solution with different NH_4_Cl concentrations (0.00, 0.10, 0.20, 0.30, 0.40, and 0.50 μg mL^−1^). The background is corrected with a blank solution (as shown in Fig. S1).

An ion-selective electrode meter (Orion Star A214 Benchtop pH/ISE Meter; Thermo Scientific) for NH_3_ detection was also performed to further verify the reliability of the colorimetric method. The details were according to our previous work [46] (as shown in Supporting Infromation).

### Determination of Hydrazine

The yield of hydrazine in the electrolyte was evaluated via Watt and Chrisp method. The hydrazine chromogenic reagent is a mixture of para-(dimethylamino) benzaldehyde (0.599 g), ethanol (300 mL) and concentrated HCl (30 mL). After 2 h ENRR reaction, 2 mL of the above reagent was added into 2 mL of the electrolyte and then the mixture was detected at 460 nm. The concentration-absorbance curve was calibrated using standard hydrazine hydrate solution with a serious of concentrations (*Y* = 2.225 *X* + 0.03, *R*^2^ = 0.99913) (as shown in Fig. S2).

### Calculation of Faradaic Efficiency (FE) and NH_3_ Yield Rate

The FE for NH_3_ production and NH_3_ yield rate were calculated at an applied potential as follow (Eqs. 1 and 2):

1 $$ {\text{FE}}_{{{\text{NH}}_{3} }} = \, C_{{{\text{NH}}_{3} }} \times V \times 3F/Q $$

2 $$ {\text{NH}}_{3} \;{\text{yield}}\;{\text{rate }} = \, C_{{{\text{NH}}_{3} }} \times V/(t \times m) $$

where *C*_NH3_ is the concentration of NH_3_, *V* is the volume of the electrolyte, *F* is the Faraday constant of 96,485 C mol^−1^, *Q* is the total charge passed through the electrochemical system, *t* is the reaction time of ENRR process, and *m* is the mass of the catalytic active site, i.e., the Bi atoms of Bi_2_O_3_ molecule on the surface of the Bi_2_O_3_/FEG electrode.

### Calculation of Equilibrium Potential

At the very beginning of the ENRR, the newly produced ammonia formed a type of coordination with water, as shown in Eq. 3:

3 $$ {\text{N}}_{2} \left( g \right) \, + \, 2{\text{H}}_{2} {\text{O }}\left( l \right) \, + \, 6{\text{H}}^{ + } \left( {{\text{aq}}.} \right) \, + \, 6 \, e^{ - } \rightleftharpoons 2{\text{ NH}}_{3} \cdot{\text{H}}_{2} {\text{O }}\left( {{\text{aq}}.} \right) $$

The equilibrium potential for ENRR under our experimental conditions is calculated using the Nernst equation as shown in Eq. 4, assuming 1 atm of N_2_ and an NH_3_·H_2_O concentration of 0.5 × 10^−5^ M in the solution [46].

4 $$ E = E^{\text{o}} - \frac{RT}{nF}\ln \left( {\frac{{\left[ {{\text{NH}}_{3} \cdot {\text{H}}_{2} {\text{O}}} \right]^{2} }}{{\left[ {{\text{H}}^{ + } } \right]^{6} }}} \right) + 0.059\,{\text{V}} \times {\text{pH}} = 0.196\,{\text{V}} ({\text{vs}}.\, {\text{RHE}}) $$

where *E*^o^ = 0.092 V is the standard potential for the above half reaction of ENRR, *R* = 8.314 J mol^−1^ K^−1^ is the molar gas constant, *n* = 6 is the number of electrons transferred in Eq. 3, *F* = 96,485 C mol^−1^ is the Faraday constant, *T* = 298.15 K is the reaction temperature in this experimental condition.

## Results and Discussion

### Materials Characterization

**Scheme 1:**
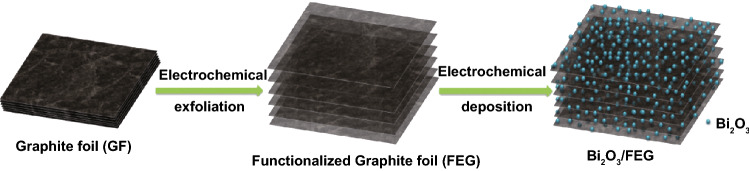
Illustration of the synthesis of Bi_2_O_3_/FEG

Scheme 1 illustrates the synthesis procedure to obtain the free-standing Bi_2_O_3_/FEG hybrid. Typically, the FEG substrate was prepared through electrochemical exfoliating the graphite foil into functionalized exfoliated graphene nanosheets with abundant oxygen functional groups. Sequentially, the Bi_2_O_3_ nanoplates were uniformly immobilized onto the FEG with BiCl_3_ as the precursor through a facile electrochemical deposition method, during which the mass loading and the morphology of the Bi_2_O_3_ can be easily controlled by adjusting the electrolyte concentration and deposition duration. The actual weight of Bi in Bi_2_O_3_/FEG electrodes was determined to be approximately 0.74 mg cm^−2^ by measuring the weight before and after the electrodeposition process, followed by ICP-AES confirmation.

**Fig. 1:**
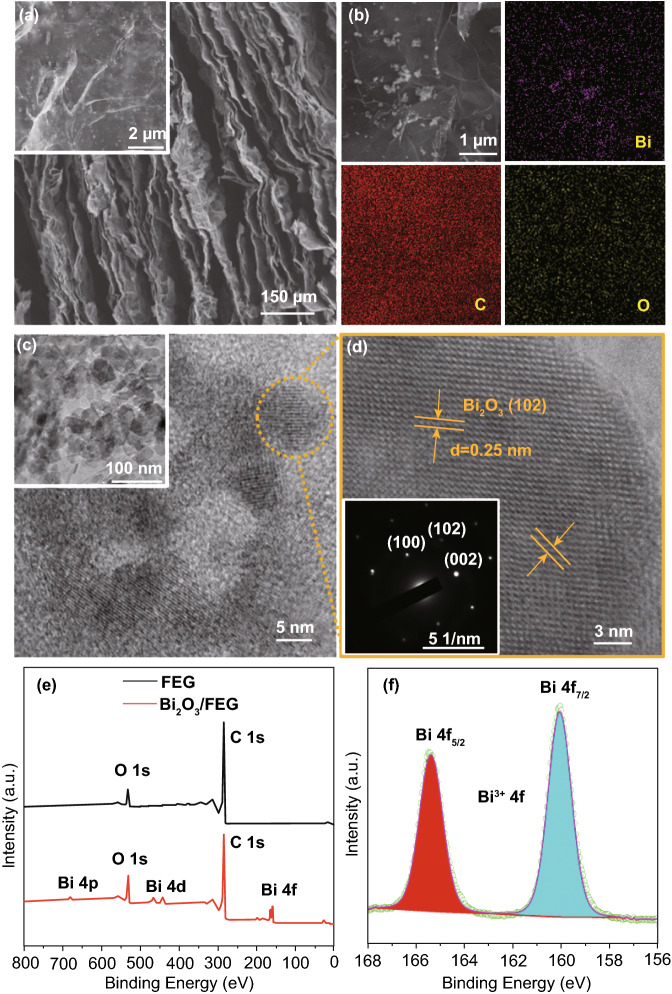
**a** SEM images of Bi_2_O_3_/FEG with the inset showing the high-magnification SEM image. **b** SEM and corresponding elemental (Bi, C, and O) mapping images of Bi_2_O_3_/FEG. **c** HRTEM images of Bi_2_O_3_/FEG, the inset shows the TEM image. **d** Corresponding HRTEM images in the circle area, the inset shows the SAED pattern. **e** XPS spectra of Bi_2_O_3_/FEG and the FEG substrate; inset is the magnified image. **f** Bi 4*f* XPS spectrum of Bi_2_O_3_/FEG

The morphology and microstructure of as-prepared Bi_2_O_3_/FEG were investigated by scanning electron microscopy (SEM) and transmission electron microscopy (TEM). Figure 1a shows the SEM images of the as-prepared Bi_2_O_3_/FEG hybrid. It can be clearly observed that, after electrochemical deposition, Bi_2_O_3_ nanoplates are uniformly grown on the surface of the loosely packed FEG layers. This is due to the fact that oxygen functional groups in graphene nanosheets play a structure-directing role in the electrochemical deposition. The Bi^3+^ precursors can be captured through coordination bonding with the hydroxyl and carboxyl groups in graphene nanosheets, which were introduced in the electrochemical exfoliation process [38]. Then this uniform deposition of Bi_2_O_3_ in the nanosheets of FEG can be achieved by the following electrochemical process. The SEM and corresponding elemental (Bi, C, and O) mapping images of Bi_2_O_3_/FEG are shown in Fig. 1b. Uniform distribution of bismuth (from Bi_2_O_3_ nanoplates) combined with the carbon and oxygen can be detected over the whole area of the Bi_2_O_3_/FEG nanosheet, further demonstrating the presence of homogeneous Bi_2_O_3_ nanoplates on the graphene sheet of FEG. The transmission electron microscopy (TEM) of the as-prepared Bi_2_O_3_/FEG hybrid is shown in Fig. 1c, which further confirms the Bi_2_O_3_ nanoplates with a particle size of about 5 nm evenly decorated on the graphene nanosheets of FEG. The TEM image of Bi_2_O_3_/FEG in Fig. 1c (inset) further displays that numerous Bi_2_O_3_ nanoplates are homogeneously dispersed on the surface of FEG. As shown in Fig. 1c, the high-resolution TEM (HRTEM) image of Bi_2_O_3_/FEG hybrid reveals the uniform distributed Bi_2_O_3_ on the surface of FEG with well-defined nanocrystalline nature. The magnification corresponding HRTEM images in the circle area of Fig. 1c reveal the lattice fringes of 0.25 nm (Fig. 1d), which could be well indexed to the (102) plane of Bi_2_O_3_. The selected area electron diffraction (SAED) pattern indicates the monocrystalline nature of Bi_2_O_3_ nanoplates and reveals the (102), (100), and (002) planes of Bi_2_O_3_ (inset of Fig. 1d), further confirming the existence of Bi_2_O_3_ in FEG.

X-ray diffraction (XRD) patterns of pure FEG and the as-prepared Bi_2_O_3_/FEG hybrid are displayed in Fig. S3. The strong peak at 26.3° of FEG is indexed to the (002) plane of graphene [36] that confirms the successful synthesis of pure FEG. It is found that Bi_2_O_3_/FEG hybrid also exhibited a characteristic peak of graphene at 26.6°. This intense and sharp peak indicates highly crystalline graphene nature of Bi_2_O_3_/FEG, which is beneficial to the electron transfer. No characteristic diffraction peaks of Bi_2_O_3_ are observed because of its lower loading content. This also implies the good dispersion of the very small Bi_2_O_3_ particles on the FEG.

The full-range X-ray photoelectron spectroscopy (XPS) spectra of FEG and the as-prepared Bi_2_O_3_/FEG hybrid are shown in Fig. 1e, and it was observed that two peaks appeared at 285.6 and 532.0 eV, corresponding to the C 1*s* and O 1*s* core level spectrum, respectively. Moreover, the presence of Bi electrons was confirmed by the XPS analysis of Bi_2_O_3_/FEG, the three doublet peaks at around 690.0, 455.0, and 162.0 eV, corresponding to the Bi 4*p*, Bi 4*d*, and Bi 4*f* electrons, indicating that some Bi_2_O_3_ was immobilized on the FEG. In the high-solution Bi 4*f* XPS spectrum (Fig. 1f), two main peaks centered at 159.8 and 165.1 eV can be identified as Bi 4*f*_7/2_ and Bi 4*f*_5/2_ signals of Bi^3+^, respectively, in accordance with previous reports [47]. And the 0.8 eV blueshift of Bi 4*f*_7/2_ and Bi 4*f*_5/2_ peaks due to charge transfer from Bi^3+^ center to the FEG. The results from the above characterizations demonstrate that FEG is an excellent substrate for the deposition and fixation of Bi_2_O_3_ nanoplates. The incorporation of Bi_2_O_3_ nanoplates into the FEG prevented the aggregation of Bi_2_O_3_ nanoplates and provided more reactive sites, that may improve its performance of ENRR.

The contact angle measurement was then performed to investigate the hydrophilicity of FEG and Bi_2_O_3_/FEG. It can be seen from Fig. S4a, the surface of FEG whose contact angle is approximately 83° exhibits strong hydrophilicity, which is mainly due to the oxygen functional groups (–OOH and –OH) of FEG. After decorated with Bi_2_O_3_, the contact angle of the as-prepared Bi_2_O_3_/FEG increases to around 110° (Fig. S4b), indicating a hydrophobic property. This hydrophobicity is expected to be helpful in promoting the ENRR performance by providing strong interaction with N_2_ gas [48].

### Electrochemical Nitrogen Reduction

**Fig. 2:**
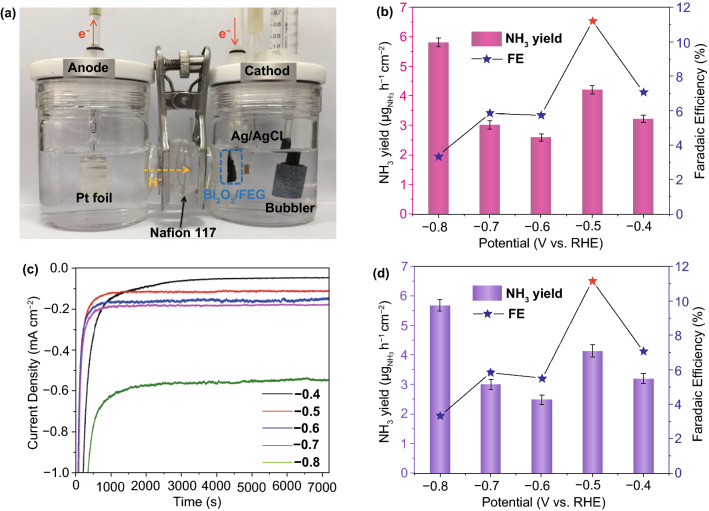
**a** Schematic reaction cell for ENRR. **b** Faradic efficiency and NH_3_ yield rate at various potentials in 0.1 M Na_2_SO_4_ electrolyte (determined by the Nessler’s reagent). **c** Chronoamperometry results at the corresponding potentials. **d** FE and NH_3_ yield rate at various potentials in 0.1 M Na_2_SO_4_ electrolyte (determined by ion-selective electrode meter)

The ENRR performance of the prepared Bi_2_O_3_/FEG catalyst was evaluated in N_2_-saturated 0.1 M Na_2_SO_4_ electrolyte under ambient conditions using a two-compartment electrochemical cell, separated by a Nafion 117 membrane. The free-standing Bi_2_O_3_/FEG hybrid material was directly used as the working electrode, with a platinum plate as the counter electrode and Ag/AgCl as the reference electrode (configuration shown in Fig. 2a). The feeding gas (ultrahigh purity N_2_, 99.999%) was continuously bubbled into the cathode via a bubbler during the experiment at the flow rate of 10 mL min^−1^. All potentials were reported on a reversible hydrogen electrode (RHE) scale. First, we studied the potential dependence of ENRR activity of Bi_2_O_3_/FEG by LSV in Ar- and N_2_-saturated 0.1 M Na_2_SO_4_ electrolytes at a scan rate of 5 mV s^−1^. As shown in Fig. S5, the LSV curves of Bi_2_O_3_/FEG in Ar and N_2_-saturated electrolytes exhibit the same shape, but a higher current density is achieved in the N_2_-saturated electrolyte when potential is more negative than − 0.4 V, implying that Bi_2_O_3_/FEG possesses catalytic activity for ENRR reaction.

Further, chronoamperometry tests at a series of potentials were carried out to explore the catalytic activity and identify the optimal potential of the Bi_2_O_3_/FEG electrode for the ENRR (Fig. 2b). The concentration of the produced ammonia in solution after the 2 h electrolysis was determined independently by a spectrophotometry method with Nessler’s reagent as a color reagent, while the possible N_2_H_4_ by-product was detected by a spectrophotometry method with *p*-C_9_H_11_NO as an indicator.

The average ammonia yields and the corresponding FE of Bi_2_O_3_/FEG at potentials from − 0.4 to − 0.8 V (vs. RHE) in 0.1 M Na_2_SO_4_ are plotted in Fig. 2b. The FE value at − 0.4 V (vs. RHE) is 7.1%, and then the ENRR rate and FE rise with the negative potential increasing until − 0.5 V (vs. RHE), where the relatively high NH_3_ yield and the maximum FE value can reach 4.21 ± 0.14 $\upmu{\text{g}}_{{{\text{NH}}_{3} }}$ h^−1^ cm^−2^ (i.e., 5.68 $\upmu{\text{g}}_{{{\text{NH}}_{3} }}$ mg_Bi_^−1^ h^−1^) and 11.2%, respectively. No N_2_H_4_ by-product has been detected, indicating the good selectivity of the Bi_2_O_3_/FEG catalyst for ENRR. Meanwhile, ENRR was also conducted on FEG in N_2_-saturated 0.1 M Na_2_SO_4_ solution at − 0.5 V (vs. RHE) to verify the source of NH_3_. No NH_3_ was detected, indicating that FEG had no catalytic activity toward ENRR for the production of NH_3_, and the contribution of NH_3_ yield from the impurities of FEG was also negligible. Therefore, it can be inferred here that the Bi_2_O_3_ in Bi_2_O_3_/FEG played a decisive role in the ENRR, while FEG possessed no activity toward the ENRR.

This ENRR catalytic performance of the as-prepared Bi_2_O_3_/FEG hybrid material can even comparable to the yields and efficiencies catalyzed by metal-based nanocatalysts as shown in Table S1. For example, Bi nanosheets (FE of 10.46% with an NH_3_ yield rate of 2.54 μg h^−1^ cm^−2^ at − 0.8 V (vs. RHE) in 0.1 M Na_2_SO_4_ electrolyte) [31] and Au nanorod (FE of 4.02% with an NH_3_ yield rate of 1.648 μg h^−1^ cm^−2^ at − 0.2 V vs. RHE in 0.1 M KOH electrolyte) [49]. The excellent ENRR performance of the as-prepared Bi_2_O_3_/FEG hybrid material is mainly due to the following reasons. On the one hand, this high ENRR activity is primarily attributed to the Bi center from Bi_2_O_3_, which can effectively inhibit the side reaction of HER during ENRR process by binding ^*^N_2_H more strongly without affecting the binding energy of ^*^NH_2_ or ^*^NH [26, 31], and offer a lower free-energy change than traditional transition mental for a better ENRR activity [29]. On the other hand, the nanosized Bi_2_O_3_ and the large surface of FEG, which provide abundant active sites, may insure the efficient ENRR reaction. Besides, the facile electron and mass transfer process from the graphene nanosheets of Bi_2_O_3_/FEG accelerate the ENRR process through promoting the electron transport, and the hydrophobic property of Bi_2_O_3_/FEG provides strong interaction with N_2_ gas further accelerate the ENRR.

In addition, as shown in Fig. 2c, the current density at different potentials behaves good stability, which can be attributed to the well dispersion and anchoring of the nanosized Bi_2_O_3_ nanoplates on the graphene nanosheets. Unexpectedly, though the NH_3_ yield reaches the highest average value of 5.8 ± 0.15 $\upmu{\text{g}}_{{{\text{NH}}_{3} }}$ h^−1^ cm^−2^ at − 0.8 V (vs. RHE), the FE values decrease obviously when the applied potentials are more negative than − 0.5 V (vs. RHE). A plausible explanation is that the ENRR in this system was in a N_2_ diffusion-controlled mode, and the feeding gas N_2_ has extremely low solubility in aqueous electrolyte. At higher negative potentials, the competing reaction (HER) was dominant [46], so the surface of Bi_2_O_3_/FEG was mainly occupied by the hydrogen molecules which would impede the mass transfer of N_2_ to the surface of Bi_2_O_3_/FEG, and thus strongly reduce the ENRR selectivity.

The concentrations of the NH_3_ synthesized by Bi_2_O_3_/FEG at potentials from − 0.4 to − 0.8 V (vs. RHE) in 0.1 M Na_2_SO_4_ were also determined by ion-selective electrode meter to confirm the reliability of this colorimetric method for ammonia detection. As shown in Fig. 2d, the corresponding average NH_3_ yields and FE were almost identical to that of determined by the Nessler’s reagent (Fig. 2b) within experimental error, suggesting that it was reliable to use the Nessler’s reagent for the quantitative analysis of the produced NH_3_.

**Fig. 3:**
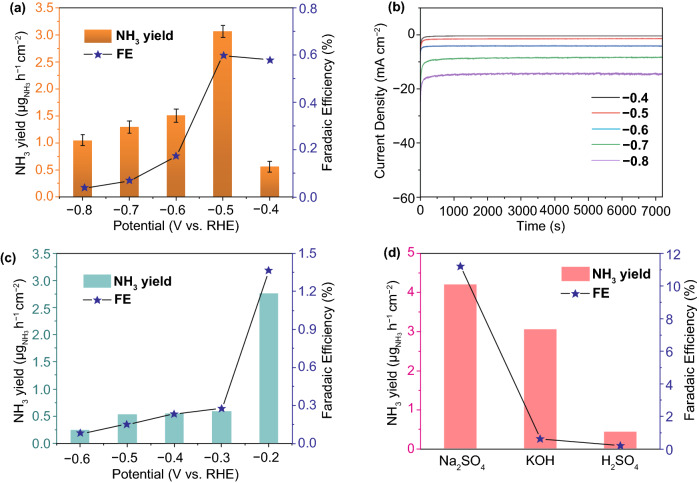
**a** FE and NH_3_ yield rate at various potentials in 0.1 M KOH electrolyte (determined by the Nessler’s reagent). **b** Chronoamperometry results at the corresponding potentials. **c** FE and NH_3_ yield rate at various potentials in 0.05 M H_2_SO_4_ electrolyte (determined by the Nessler’s reagent). **d** Optimum FE and yield rates of NH_3_ in different electrolytes (at − 0.5 V vs. RHE, determined by the Nessler’s reagent)

Subsequently, chronoamperometry tests in N_2_-saturated 0.1 M KOH or 0.05 M H_2_SO_4_ electrolyte under the same experimental conditions as that of in Na_2_SO_4_ electrolyte were carried out to explore the catalytic activity of the Bi_2_O_3_/FEG in strong basic and acidic media. As shown in Fig. 3a, the optimum average NH_3_ yield rate 3.07 ± 0.11 $\upmu{\text{g}}_{{{\text{NH}}_{3} }}$ h^−1^ cm^−2^ in thestrong basic electrolyte occurred at − 0.5 V (vs. RHE), indicating a wide pH response of Bi_2_O_3_/FEG. However, the relatively low FE of 0.59% may be caused by the high current density implying dominant HER (Fig. S7). Furthermore, the robust current stability of Bi_2_O_3_/FEG in 0.1 M KOH has been confirmed for 2 h electrolysis, as shown in Fig. 3b, indicating ideal stability of Bi_2_O_3_/FEG in the strong basic electrolyte. The NH_3_ yields and the corresponding FE of Bi_2_O_3_/FEG at potentials from -0.2 to -0.6 V (vs. RHE) in 0.05 M H_2_SO_4_ are plotted in Fig. 3c. The NH_3_ yield at − 0.2 V (vs. RHE) is 2.8 $\upmu{\text{g}}_{{{\text{NH}}_{3} }}$ h^−1^ cm^−2^ with the FE of 1.36%, then the NRR rate and FE decrease sharply with the potential shifted negatively. The dissatisfactory catalytic performance in 0.05 M Na_2_SO_4_ electrolyte should attribute to the instability of Bi_2_O_3_ and the drastic HER competing reaction in acid medium. Comparing the optimum FE and NH_3_ yield rates of Bi_2_O_3_/FEG in different electrolytes as shown in Fig. 3d, Bi_2_O_3_/FEG exhibits excellent electrocatalysis performance and stability for ENRR in neutral media even better than that in strong basic and acidic media under the same ambient conditions. It is highly desired to develop electrocatalysts for efficient and durable ENRR catalysis at neutral conditions, which is beneficial for alleviating the serious environment sufferings, reducing equipment and catalyst corrosions, lower the cost of the whole ENRR process, and further accelerating the practical applications of ENRR.

**Fig. 4:**
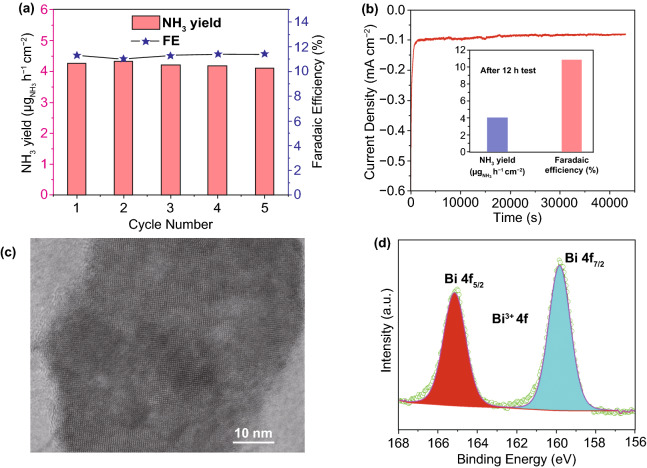
**a** FE and NH_3_ yield rates of Bi_2_O_3_/FEG calculated after consecutive recycling electrolysis in N_2_-saturated 0.1 M K_2_SO_4_ solution at − 0.5 V (vs. RHE) for 2 h. **b** 12 h durability test for Bi_2_O_3_/FEG toward ENRR at − 0.5 V (vs. RHE), inset: the corresponding FE and NH_3_ yield rate after durability test. **c** The HRTEM image of Bi_2_O_3_/FEG after ENRR. **d** Bi 4*f* XPS spectrum of Bi_2_O_3_/FEG after ENRR

The stability of the Bi_2_O_3_/FEG was evaluated by consecutive recycling electrolysis and long-term chronoamperometry test at − 0.5 V (vs. RHE) in 0.1 M Na_2_SO_4_. As shown in Fig. 4a, no obvious change in the NH_3_ yield and Faradaic efficiency could be observed during five consecutive recycling electrolysis, which suggests the excellent stability of Bi_2_O_3_/FEG for NH_3_ synthesis at ambient conditions. In addition, slight degradation of current density in the process of long-term chronoamperometry test is detected for 12 h at − 0.5 V (vs. RHE) (Fig. 4b). Moreover, no obvious variation of the NH_3_ yield rate and FE could be observed after 12 h long-term chronoamperometry test, indicating the high robustness of Bi_2_O_3_/FEG toward ambient NH_3_ synthesis. Furthermore, both the compositions and Bi_2_O_3_ nature of the Bi_2_O_3_/FEG could be well maintained after the long-term electrocatalysis reaction when comparing TEM images (Fig. 4c) and XPS (Fig. 4d) before and after the reaction. All these observations demonstrate the excellent electrochemical durability of the Bi_2_O_3_/FEG for the ENRR, which is another crucial factor for the enhancement of ENRR performances.

## Conclusions

In summary, a highly efficient hybrid ENRR electrocatalyst composed of nanosized Bi_2_O_3_ supported on FEG was developed. Due to the synergistic effect between the nanosized Bi_2_O_3_ and the large surface area of FEG, the Bi_2_O_3_/FEG hybrid exhibited the superb electrocatalytic performance toward ENRR at a low overpotential of − 0.5 V (vs. RHE) in the neutral electrolyte and under ambient temperature and pressure. The FE of Bi_2_O_3_/FEG in 0.1 M Na_2_SO_4_ electrolyte is 11.2% with the NH_3_ yields of 5.68 $\upmu{\text{g}}_{{{\text{NH}}_{3} }}$ mg_Bi_^−1^ h^−1^, which is superior to that of most state-of-the-art ENRR electrocatalysts reported up to date. The high ENRR activity is attributed to the Bi center from Bi_2_O_3_ during the ENRR process with the help of the facile electron transfer process from the graphene nanosheets of FEG. This work opens an avenue to rational designing of Bi-based carbon hybrid electrocatalysts and highlights the catalytic center engineering strategy to effectively manipulate the catalytic performance of electrocatalysts.

## Electronic supplementary material

Below is the link to the electronic supplementary material.

Supplementary material 1 (PDF 559 kb)
