# Supplementary material for: Bismuth-Based Free-Standing Electrodes for Ambient-Condition Ammonia Production in Neutral Media
Source: Nanomicro Lett. 2020 Jun 19;12:133. doi: 10.1007/s40820-020-00444-y (PMC7770657; doi:10.1007/s40820-020-00444-y)
Supplement: Supplementary file 1 — Supplementary material 1 (PDF 559 kb) [file 40820_2020_444_MOESM1_ESM.pdf]

Supporting Information for

Bismuth-Based Free-Standing Electrodes for Ambient-Condition Ammonia Production in Neutral Media

Ying Sun[1, 2], Zizhao Deng[1], Xi-Ming Song[3], Hui Li[1], Zihang Huang[1], Qin Zhao[1], Daming Feng[1], Wei Zhang[3], Zhaoqing Liu[4], Tianyi Ma[2, *]

[1]Institute of Clean Energy Chemistry, Key Laboratory for Green Synthesis and Preparative Chemistry of Advanced Materials, College of Chemistry, Liaoning University, Shenyang 110036, People's Republic of China

[2]Discipline of Chemistry, University of Newcastle, Callaghan, NSW 2308, Australia

[3]Key Laboratory for Green Synthesis and Preparative Chemistry of Advanced Materials, College of Chemistry, Liaoning University, Shenyang 110036, People's Republic of China

[4]School of Chemistry and Chemical Engineering, Guangzhou Key Laboratory for Environmentally Functional Materials and Technology, Guangzhou University, Guangzhou 510006, People's Republic of China

*Corresponding author. E-mail: Tianyi.Ma@newcastle.edu.au (Tianyi Ma)

S1 Materials

Bismuth Chloride ($BiCl_3$, ≥ 99.99%), Ammonium chloride (NH_4Cl, ≥99.99%), Nessler's reagent, para-(dimethylamino) benzaldehyde (99%) are purchased from Sigma-Aldrich Chemical Reagent Co., Ltd. Potassium carbonate (Na_2CO_3, AR), Potassium nitrate ($NaNO_3$, AR), Potassium chloride (KCl, AR), Sodium potassium tartrate ($NaKC_4H_4O_6·4H_2O$, AR), Sodium sulfate anhydrous (Na_2SO_4, AR), Potassium hydroxide (KOH, AR), Hydrogen peroxide aqueous solution (H_2O_2, 30.0%) Ethylene glycol [$(CH_2OH)_2$, AR], Ethanol (C_2H_5OH, 99.5%) are purchased from Sinopharma chemical reagent co. Ltd. All chemicals were used without further purification. Graphite foil with a thickness of 0.35 mm, ρ=1 g cm^{-3} (GF). Nafion 117 membrane (Dupont) was purchased from the Fuelcell store. Ultrapure water used throughout all experiments was purified through a Millipore system (Millipore, 18.2 MΩ·cm). High purity N_2 gas (≥ 99.999 %) and Ar (≥ 99.999%) gas were provided by Shenyang Zhaote Gas Co., LTD.

S2 Characterization

The UV-vis spectra were obtained on a Shimadzu UV-2600 spectrophotometer. The

SEM images and EDX mappings were acquired using a Hitachi SU-8010 equipped with an EDX analyzer operated at an accelerating voltage of 15 kV. The TEM images were obtained on a JEM-2100 operating at 200 kV. XPS spectra were recorded by the Thermo Scientific ESCALAB250Xi spectrometer equipped with an Al Kα monochromatic. The water contact angle was measured on a HUAVE S24-100.

S3 Quantification of NH_3 by an Ion-selective Electrode Meter

First, a series of standard ammonia solutions (10, 100, 1000, and 10,000 ppb) were prepared from a stock solution (1000 ppm ammonia as nitrogen standard) for the calibration with the slope in the range of −56 to −60 mV when the standards were between 20 and 25 °C. To minimize the impact of the background of the N_2-saturated electrolyte (0.5 M NaOH) on the quantitative analysis of the produced NH_3, each standard ammonia solution was prepared by the dilution of the stock NH_4Cl solution using the N_2-saturated 0.5 M NaOH solution. Ionic strength adjuster (ISA) was used to provide a constant background ionic strength and adjust the solution pH. ISA must be added to all samples and standards immediately before measurement to prevent ammonia loss, and 80 mL of standard or sample required the addition of 1.6 mL ISA with the stirring thoroughly. In addition, to test if ammonia escaped from the electrolyte solution, the outlet gas was introduced to an acid bottle for wet scrubbing to collect the possible escaping ammonia. The ion-selective electrode meter was employed to measure the ammonia concentration in the acid wet scrubbing bottle, and the experimental results revealed that no ammonia was detected, suggesting that the ammonia escaping from the electrolyte solution could be negligible.

S4 Supplementary Figures

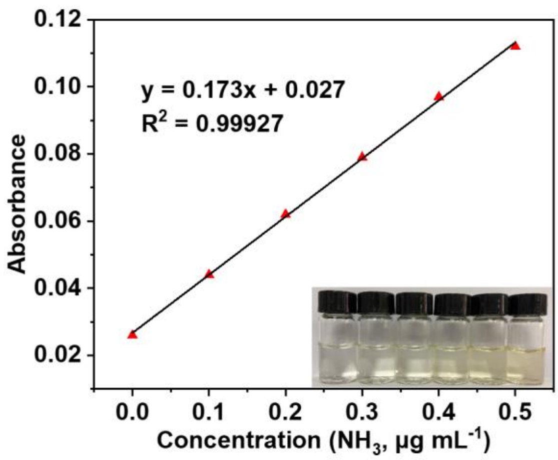

Fig. S1 The linear fit (y = ax + b) of the calibration curves using Nessler's reagent. The background of 0.1 M Na_2SO_4 aqueous solution without NH_4Cl was subtracted from all data.

The concentrations of the synthesized NH_3 in the electrolyte was measured by a colorimetric method using Nessler's reagent as the color reagent. For this method, 10 mL electrolyte was mixed with 0.2 mL 50% seignette salt solution. Then 0.2 mL

Nessler's reagent was added and the mixture was allowed still for 10 min. Then the mixture was detected as the absorbance at 420 nm by an UV-vis spectrometer (Shimadzu UV-2600). A standard curve of the Nessler's reagent-based colorimetric method is constructed by measuring a series of absorbances for the reference solution with different NH_4Cl concentrations (0.00, 0.10, 0.20, 0.30, 0.40, and 0.50 μg mL^{-1}). The back-ground is corrected with a blank solution.

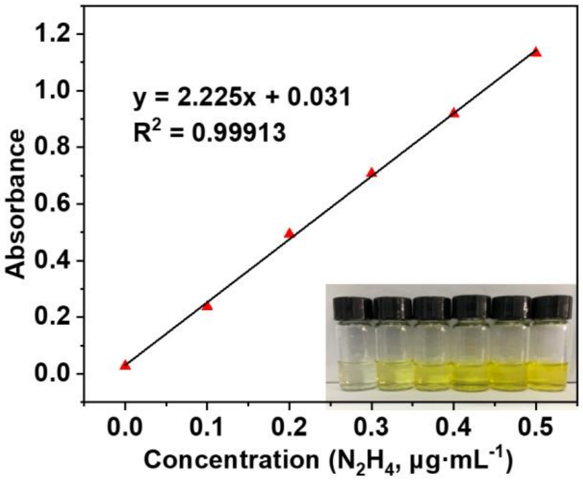

Fig. S2 Calibration curve for colorimetric N_2H_4 assay using p-$C_9H_{11}NO$. The line shows the linear fit (y = ax + b) of the calibration curves. The background of 0.1 M Na_2SO_4 aqueous solution without NH_4Cl was subtracted from all data.

The yield of hydrazine in the electrolyte was evaluated via Watt and Chrisp method. The hydrazine chromogenic reagent is a mixture of para-(dimethylamino) benzaldehyde (0.599 g), ethanol (300 mL) and concentrated HCl (30 mL). After 2 h NRR reaction, 2 mL of the above reagent was added into 2 mL of the electrolyte, then the mixture was detected at 460 nm. The concentration-absorbance curve was calibrated using standard hydrazine hydrate solution with a serious of concentrations (Y= 2.225 X + 0.03, R^2 = 0.99913).

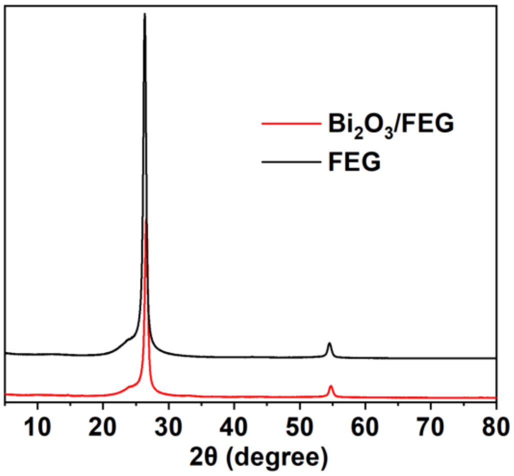

Fig. S3 XRD patterns of Bi_2O_3/FEG and FEG

X-ray diffraction (XRD) pattern of pure FEG and the as-prepared Bi_2O_3/FEG hybrid are displayed in Figure S1. The strong peak at 26.3° of FEG is indexed to the (002) plane of graphene, which confirms the successful synthesis of pure FEG. It is found that Bi_2O_3/FEG hybrid also exhibited a characteristic peak of graphene at 26.6°. This intense and sharp peak indicates highly crystalline graphene nature of Bi_2O_3/FEG, which is beneficial to the electron transfer. No characteristic diffraction peaks of Bi_2O_3 are observed because of its lower loading content, on the other hand, also implying the good dispersion of the very small Bi_2O_3 particles on the FEG.

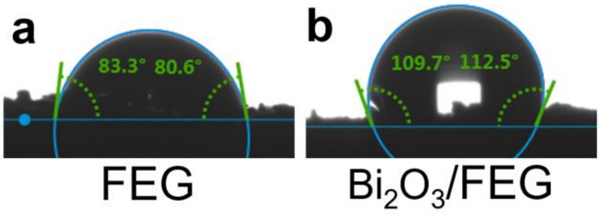

Fig. S4 Water droplet contact angle measurement on (**a**) FEG and (**b**) Bi_2O_3/FEG

The contact angle measurement was then performed to investigate the hydrophilicity of FEG and Bi_2O_3/FEG. It can be seen from Fig. S4a, the surface of FEG whose contact angle is approximate 83° exhibits strong hydrophilicity, which is mainly due to the oxygen functional groups (-OOH and -OH) of FEG. After decorated with Bi_2O_3, the contact angle of the as-prepared Bi_2O_3/FEG increases to around 110° (Fig. S4b), indicating a hydrophobic property. This hydrophobicity is expected to be helpful in promoting the ENRR performance by providing strong interaction with N_2 gas.

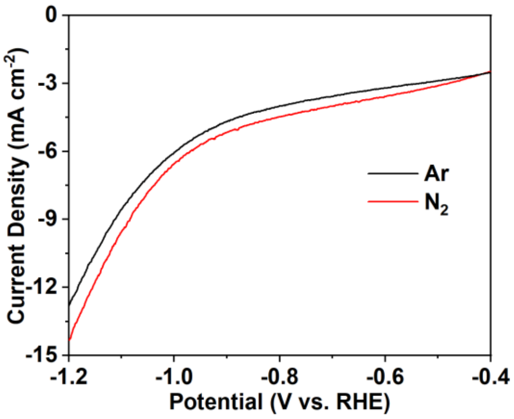

Fig. S5 LSV curves of Bi_2O_3/FEG in Ar- and N_2-saturated 0.1 M Na_2SO_4 with a scan rate of 5 mV s^{-1}

The potential dependence of ENRR activity of Bi_2O_3/FEG was studied by linear sweep voltammetry (LSV) in Ar- and N_2-saturated 0.1 M Na_2SO_4 electrolytes with a scan rate of 5 mV s^{-1}. The LSV curves have the same shape, but a higher current density is achieved in the N_2-saturated electrolyte when potential is more negative than -0.4 V, implying that Bi_2O_3/FEG possesses catalytic activity for ENRR reaction.

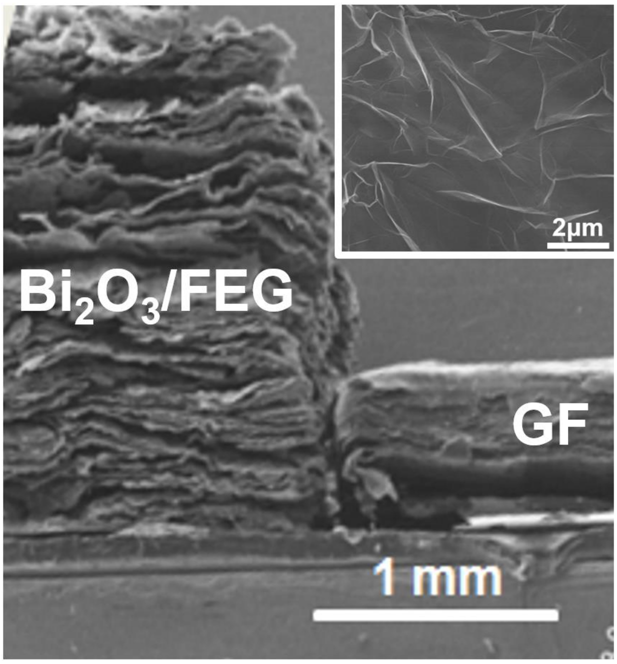

Fig. S6 SEM images of Bi_2O_3 /FEG and GF, the inset showing the high magnification SEM image of FEG

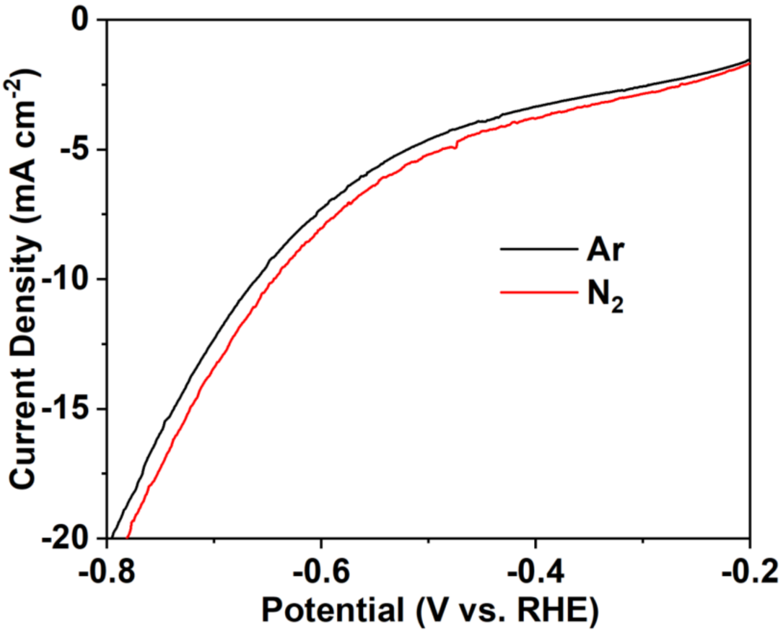

Fig. S7 LSV curves of Bi_2O_3/FEG in Ar- and N_2-saturated 0.1 M NaOH with a scan rate of 5 mV s^{-1}

The potential dependence of ENRR activity of Bi_2O_3/FEG was studied by linear sweep voltammetry (LSV) in Ar- and N_2-saturated 0.1 M NaOH electrolytes with a scan rate of 5 mV s^{-1}. The LSV curves have the same shape, but a higher current density is achieved in the N_2-saturated electrolyte when potential is more negative than -0.2 V, implying that Bi_2O_3/FEG possesses catalytic activity for ENRR reaction.

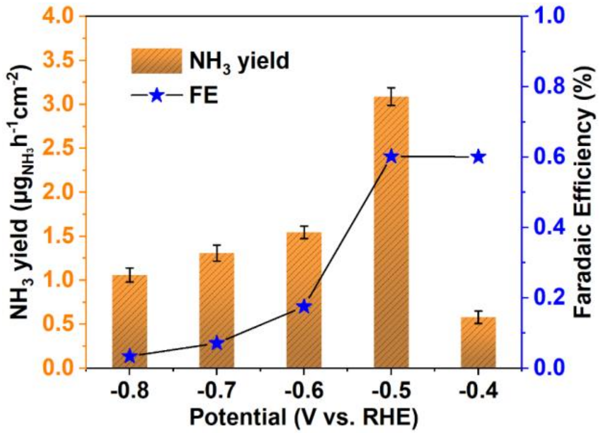

Fig. S8 Faradic efficiency and NH_3 yield rate at various potentials in 0.1 M NaOH electrolyte (determined by ion-selective electrode meter)

The concentrations of the NH_3 synthesized by Bi_2O_3/FEG at potentials from -0.4 to -0.8 V (vs. RHE) in 0.1 M NaOH were also determined by ion-selective electrode meter to confirm the reliability of this colorimetric method for ammonia detection. As shown in Fig. S8, the corresponding average NH_3 yields and FE were almost identical to that of determined by the Nessler's reagent (in Fig. 3a) within experimental error, suggesting that it was reliable to use the Nessler's reagent for the quantitative analysis of the produced NH_3.

Table 1 Comparison of the ENRR electrocatalytic activity of Bi_2O_3/FEG and other metal-based nano-catalysts at ambient condition

**Catalyst**	**Electrolyte**	**NH_3 Yield Rate**	**Potential (V vs. RHE)**	**FE (%)**	**Reference**
Bi_2O_3@FEG	0.1 M Na_2SO_4	4.21 μg h^{-1} cm^{-2}	-0.5	11.2	**This work**
Nb_2O_5/CC	0.1 M Na_2SO_4	1.58×10^{-10} mol s^{-1} cm^{-2}	-0.6	2.26	[S1]
NiCoS/C nanocages	0.1 M Li_2SO_4	26.0 μg h^{-1} $mg^{-1}_{cat.}$	0	12.9	[S2]
AuNPs@MoS_2	0.1 M Na_2SO_4	5.65 μg h^{-1} $mg^{-1}_{cat.}$	-0.3	9.7	[S3]
Bi NS	0.1 M Na_2SO_4	2.54 μg h^{-1} cm^{-2}	-0.8	10.46	[S4]
Defect-rich MoS_2	0.1 M Na_2SO_4	29.28 μg h^{-1} $mg^{-1}_{cat.}$	-0.4	8.34	[S5]
MoS_2	0.1 M Na_2SO_4	8.08×10^{-11} mol s^{-1} cm^{-2}	-0.5	1.17	[S6]
Cr_2O_3 microsphere	0.1 M Na_2SO_4	25.3 μg h^{-1} $mg^{-1}_{cat.}$	-0.9	6.78	[S7]
Nb_2O_5 nanofiber	0.1 M HCl	43.6 μg h^{-1} $mg^{-1}_{cat.}$	-0.55	9.26	[S8]
Au_1Cu_1	0.05 mol L^{-1} H_2SO_4	154.91 μg h^{-1} $mg^{-1}_{cat.}$	-0.2	54.96	[S9]
Co_3Fe–MOF	0.1 M KOH	8.79 μg h^{-1} $mg^{-1}_{cat.}$	-0.2	25.64	[S10]

Bi nanosheets	0.1 M $NaHCO_3$	12.49 μg h^{-1} $mg^{-1}_{cat.}$	-0.3	7.09	[S11]
Mn_3O_4	0.1 M Na_2SO_4	11.6 μg h^{-1} $mg^{-1}_{cat.}$	-0.8	3.0	[S12]
Au NPs	0.1 M KOH	17.49 μg h^{-1} $mg^{-1}_{cat.}$	-0.14	5.79	[S13]
TiO_2–rGO	0.1 M Na_2SO_4	15.13 μg h^{-1} $mg^{-1}_{cat.}$	-0.9	3.3	[S14]
TiO_2	0.1 M Na_2SO_4	9.16×10^{-11} mol s^{-1} cm^{-2}	-0.7	2.5	[S15]
Au nanorod	0.1 M KOH	1.648 μg h^{-1} cm^{-2}	-0.2	4.02	[S16]
FeS@MoS_2/CFC	0.1 M Na_2SO_4	8.45 μg h^{-1} cm^{-2}	-0.5	2.96	[S17]
γ-Fe_2O_3	0.1 M KOH	12.5 nmol h^{-1} $mg^{-1}_{cat.}$	0	1.9	[S18]

Supplementar References

[S1]W.H. Kong, Z.C. Liu, J.R. Han, L. Xia, Y. Wang et al., Ambient electrochemical N_2-to-NH_3 fixation enabled by Nb_2O_5 nanowire array. Inorg. Chem. Fronties **2**, 423-427 (2019). http://dx.doi.org/10.1039/C8QI01049H

[S2]X.K. Wu, Z.C. Wang, Y. Han, D. Zhang, M.H. Wang et al., Chemically coupled NiCoS/C nanocages as efficient electrocatalysts for nitrogen reduction reactions. J. Mater. Chem. A **2**, 543-547 (2020). http://dx.doi.org/10.1039/C9TA10142J

[S3]Y. Zhou, X.P. Yu, X.Y. Wang, C. Chen, S.T. Wang, J. Zhang, MoS_2 nanosheets supported gold nanoparticles for electrochemical nitrogen fixation at various pH value. Electrochim. Acta 34-41 (2019). https://doi.org/10.1016/j.electacta.2019.05.111

[S4]L.Q. Li, C. Tang, B.Q. Xia, H.Y. Jin, Y. Zheng, S.Z. Qiao, Two-dimensional mosaic bismuth nanosheets for highly selective ambient electrocatalytic nitrogen reduction. ACS Cataly. **4**, 2902-2908 (2019). https://doi.org/10.1021/acscatal.9b00366

[S5]X.H. Li, T.S. Li, Y.J. Ma, Q. Wei, W.B. Qiu et al., Boosted electrocatalytic N_2 reduction to NH_3 by defect-rich MoS_2 nanoflower. Adv. Energy Mater. **30**, 1801357 (2018). https://doi.org/10.1002/aenm.201801357

[S6]L. Zhang, X.Q. Ji, X. Ren, Y.J. Ma, X.F. Shi et al., Electrochemical ammonia synthesis via nitrogen reduction reaction on a MoS_2 catalyst: theoretical and experimental studies. Adv. Mater. **28**, 1800191 (2018). https://doi.org/10.1002/adma.201800191

[S7]Y. Zhang, W.B. Qiu, Y.J. Ma, Y.L. Luo, Z.Q. Tian et al., High-performance electrohydrogenation of N_2 to NH_3 catalyzed by multishelled hollow Cr_2O_3 microspheres under ambient conditions. ACS Cataly. **9**, 8540-8544 (2018). https://doi.org/10.1021/acscatal.8b02311

[S8]J.R. Han, Z.C. Liu, Y.J. Ma, G.W. Cui, F.Y. Xie et al., Ambient N_2 fixation to NH_3 at ambient conditions: using Nb_2O_5 nanofiber as a high-performance

electrocatalyst. Nano Energy 264-270 (2018). https://doi.org/10.1016/j.nanoen.2018.07.045

[S9]Y.Q. Liu, L. Huang, X.Y. Zhu, Y.X. Fang, S.J. Dong, Coupling Cu with Au for enhanced electrocatalytic activity of nitrogen reduction reaction. Nanoscale **3**, 1811-1816 (2020). http://dx.doi.org/10.1039/C9NR08788E

[S10] W.X. Li, W. Fang, C. Wu, K.N. Dinh, H. Ren, L. Zhao, C.T. Liu, Q.Y. Yan, Bimetal–MOF nanosheets as efficient bifunctional electrocatalysts for oxygen evolution and nitrogen reduction reaction. J. Mater. Chem. A **7**, 3658-3666 (2020). http://dx.doi.org/10.1039/C9TA13473E

[S11] L. Xia, W.Z. Fu, P.Y. Zhuang, Y.D. Cao, M.O.L. Chee, P. Dong, M.X. Ye, J.F. Shen, Engineering abundant edge sites of bismuth nanosheets toward superior ambient electrocatalytic nitrogen reduction via topotactic transformation. ACS Sustain. Chem. Eng. **7**, 2735-2741 (2020). https://doi.org/10.1021/acssuschemeng.9b06449

[S12] X.F. Wu, L. Xia, Y. Wang, W.B. Lu, Q. Liu, X.F. Shi, X.P. Sun, Mn_3O_4 nanocube: an efficient electrocatalyst toward artificial N_2 fixation to NH_3. Small **48**, 1803111 (2018). https://doi.org/10.1002/smll.201803111

[S13] C. Chen, C. Liang, J. Xu, J.K. Wei, X.R. Li, Y. Zheng, J.R. Li, H.L. Tang, J.S. Li, Size-dependent electrochemical nitrogen reduction catalyzed by monodisperse Au nanoparticles. Electrochim. Acta **335**, 135708 (2020). https://doi.org/10.1016/j.electacta.2020.135708

[S14] X.X. Zhang, Q. Liu, X.F. Shi, A.M. Asiri, Y.L. Luo, X.P. Sun, T.S. Li, TiO_2 nanoparticles–reduced graphene oxide hybrid: an efficient and durable electrocatalyst toward artificial N_2 fixation to NH_3 under ambient conditions. J. Mater. Chem. A **36**, 17303-17306 (2018). http://dx.doi.org/10.1039/C8TA05627G

[S15] R. Zhang, X. Ren, X.F. Shi, F.Y. Xie, B.Z. Zheng, X.D. Guo, X.P. Sun, Enabling effective electrocatalytic N_2 conversion to NH_3 by the TiO_2 nanosheets array under ambient conditions. ACS Appl. Mater. Interfaces **34**, 28251-28255 (2018). https://doi.org/10.1021/acsami.8b06647

[S16] D. Bao, Q. Zhang, F.L. Meng, H.X. Zhong, M.M. Shi et al., Electrochemical reduction of N_2 under ambient conditions for artificial N_2 fixation and renewable energy storage using N_2/NH_3 cycle. Adv. Mater. **3**, 1604799 (2017). https://doi.org/10.1002/adma.201604799

[S17] Y.X. Guo, Z.Y. Yao, B.J.J. Timmer, X. Sheng, L.Z. Fan, Y.Y. Li, F.G. Zhang, L.C. Sun, Boosting nitrogen reduction reaction by bio-inspired FeMoS containing hybrid electrocatalyst over a wide pH range. Nano Energy **62**, 282-288 (2019). https://doi.org/10.1016/j.nanoen.2019.05.051

[S18] J.M. Kong, A. Lim, C. Yoon, J.H. Jang, H.C. Ham et al., Electrochemical synthesis of NH_3 at low temperature and atmospheric pressure using a γ-Fe_2O_3 catalyst. ACS Sustain. Chem. Eng. **11**, 10986-10995 (2017). https://doi.org/10.1021/acssuschemeng.7b02890
